# On the role of resonance in drug failure under HIV treatment interruption

**DOI:** 10.1186/1742-4682-10-44

**Published:** 2013-07-11

**Authors:** Leonardo Oña, Roger D Kouyos, Michael Lachmann, Sebastian Bonhoeffer

**Affiliations:** 1 Max Planck Institute for Evolutionary Anthropology, Deutscher Platz 6 04103 Leipzig, Germany; 2 Department of Ecology and Evolutionary Biology, Princeton University, Princeton, New Jersey, USA; 3 Institute of Integrative Biology, ETH-Zurich (Swiss Federal Institute of Technology), Zurich, Switzerland

## Abstract

**Background:**

The application of highly active antiretroviral therapy (HAART) against HIV can reduce and maintain viral load below detection limit in many patients. Continuous HAART, however, can have severe side effects. In this context, structured treatment interruptions (STI) were considered to be a promising strategy. However, using CD4 cell count to guide intermittent therapy starting and stopping points, the SMART study (strategies for management of antiretroviral therapy), revealed that STI were associated with increased risk of AIDS and other complications. Additionally, short-term periodic (e.g. one week on / one week off) interruption therapies have shown virus rebound exceeding a given “failure threshold”, without any evidence for the evolution of drug resistance. Currently, the only hypothesis explaining the failure of STI is the “resonance hypothesis”, which posits that treatment failure is due to a resonance effect between the drug treatment and the viral population. In the present study we used a mathematical model to analyse the parameters affecting the output of drug treatment interruption and the premises of the resonance hypothesis.

**Methods:**

We used a population dynamic model of HIV infection. Simulations and analytical approximations of deterministic and stochastic versions of the model were studied.

**Results and Conclusion:**

The present study examines the roles of the most important parameters affecting the viral rebound, responsible for drug failure. We related these findings to the resonance hypothesis, and showed that the degree of sustainability of damping oscillations present in the model after the acute phase is strongly linked to their amplitude, which determines the resonance level. Stochastic simulations of the same model even revealed sustained oscillations in virus population for small virus population sizes. Given that pronounced viral load oscillations have not been observed in HIV-1 patients, the link between oscillations and resonance level suggests that treatment failure due to a resonance effect is not plausible. Moreover, the failure threshold is attained before the virus population crosses the set point while growing. As the maximum virus population is reached even after the set point is crossed, the role of resonance effects in the context of treatment interruptions cannot explain drug failure.

## Background

HIV infection is characterised by an exponential increase of the virus population during a so-called acute phase, which is followed by a decrease, before reaching a (more or less fluctuating) steady state, called set point. This steady state characterises the asymptomatic phase. At this stage, the viral load can vary more than 1000-fold between patients [1]. Drug therapy often decreases the virus population to lower, almost undetectable levels [2]. When drug administration is interrupted, the virus population increases at different rates in each patient.

The combined administration of different types of drugs, known as highly active antiretroviral therapy (HAART), can reduce and maintain viral load below detection limit in many patients. The introduction of HAART methods have thereby dramatically improved the average prognosis of HIV infections. However, in addition to having potentially toxic side effects, drugs can also be inaccessible due to cost [3,4]. Structured Treatment Interruptions (STI), consisting of therapy withdrawal and re-initiation according to specific criteria, has been proposed to reduce these problems [5,6]. The protocols vary from non-periodic randomised controlled trials [7], to the most common, a periodic one week on/one week off scheme [3,4]. Although this method has shown some degree of success [3,4,8-10], an important large-scale study showed failure in half of the cases [8]. Failure occurs when the viral rebound exceeds a given “failure threshold”, and is apparently not caused by virus resistance [8]. On the other hand, by using CD4 cell count to guide intermittent therapy starting and stopping points, the National Institute of Health’s Strategies for Management of Antiretroviral Therapy (SMART) study [11], revealed that STI was associated with increased risk of AIDS or death, serious AIDS-defining events, and other severe complications.

Although STI has, in recent years, become an obsolete approach to cope with HIV infections, the investigation of the viral population dynamics after drug interruption can still offer valuable insights. Any perturbation of the system can provide information about the forces underlying the population biology of the virus, and can also shed light on current problems of HAART such as the consequences of drug interruption resulting from low drug adherence.

A prominent hypothesis states that resonance effects between virus oscillations and the administered drug treatment account for the failure of STI [12]. By studying patient-specific resonance spectra (including the maximal, minimal and average viral response to different times of drug administration and interruption), the authors found patterns in agreements with empirical observations, e.g. that two patients with similar initial set points can present different virological responses. One of the weaknesses of the resonance hypothesis is the requirement that an oscillatory behaviour should exist in the HIV population within patients. Although the authors do not specify the relationship between the resonance effect and the oscillations, they acknowledge that the resonance hypothesis is speculative because oscillations have not been observed in HIV [12].

In this study, we used the population dynamic model underlying the resonance hypothesis to derive the most important parameters affecting the viral rebound. We have classified the parameters into those that affect the viral set point, those that affect the viral rebound rate, and those that alter both. We have then studied the relationship between the amplitude of the resonance oscillations – present in the model after the acute phase – and their degree of sustainability. We have also evaluated the effect of stochasticity on the oscillations for different viral population sizes. Finally, we have analysed the relationship between the failure threshold, the viral set point, and the viral rebound rate with regard to their role in the failure of STI.

## Materials and methods

The basic model of HIV-virus dynamics [13-15] has three variables: uninfected cells, x; infected cells, y; and free virus particles, v, and is given by the following set of differential equations:

(1) $$\begin{array}{ll} \dot{x}=\lambda -\text{dx}-\mathrm{\beta xv} & \; \end{array}$$

(2) $$\begin{array}{ll} \dot{y}=\mathrm{\beta xv}-\text{ay} & \; \end{array}$$

(3) $$\begin{array}{ll} \dot{v}=\text{ky}-\text{uv} & \; \end{array}$$

Uninfected cells are produced at a constant rate, *λ*, and die at the rate *dx*. Free virus infects uninfected cells to produce infected cells at rate *βxv*. Infected cells die at rate *ay*. New virus is produced from infected cells at rate *ky* and dies at rate *uv*. Therefore, the average life-times of uninfected cells, infected cells, and free virus are thus given by 1/*d*, 1/*a*, and 1/*u*, respectively. The average number of virus particles produced over the lifetime of a single infected cell (the burst size) is given by *k*/*a*[13]. The virus variable *v*, defines the total amount of virus particles that are able to infect uninfected cells, and therefore generate cell-to-cell transmission, after a complete round of infection including proper integration in the infected cells. The basic reproductive ratio, *R*_0_, is defined as the average number of newly infected cells that arise from any one infected cell when almost all cells are uninfected. Here *R*_0_=*βλk*/(*adu*).

We performed both deterministic and stochastic simulations of this model. The stochastic simulations were performed employing an exact approximation using the Gillespie algorithm [16] and for large population sizes we used Tau Leap binomial methods [17]. The analytical equivalence between the stochastic and the deterministic formulation is presented in the Additional file 1.

## Results

### The resonance effect

In this section we reviewed the analysis from Breban and Blower [12], and summarised their results, proposing a quantitative measurement for the resonance level.

Numerical simulations of the model given by Equations (1-3) show that there can be a significant rebound of virus load after drug interruption only under certain specific conditions. Moreover, the strength of the viral rebound depends on the selected parameters. Here, each parameter combination represents a (simulated) patient [12].

**Figure 1:**
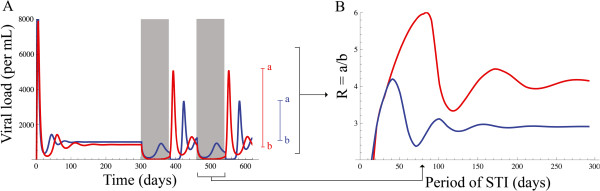
**Simulation of viral load and the effect of drug interruption over time (A) and as a function of the period of STI (B).** Shaded areas represent the intervals of time on treatment, modelled as a decrease by 30 % in *β* and *k* as in [12]. Red and blue are the patients A and B from [12]. Patient A: *λ*=20, *d*=0.02, *a*=0.4, *u*=4.0, *β*=5 10^−5^, *k*=100, and patient B, as A except *β*=8 10^−5^. The level of resonance (R) is defined by the ratio of a (the maximum value attained by the virus population) and b (the viral set point).

The “resonance spectra” plots for each patient, ( [12]; Figure two) includes the minimum, maximum, and average viral load at different periods of STI. These plots show that patients can begin with similar set points, but end up with very different maximum viral population size values after drug interruption, or the opposite: very different set points, but similar levels of the maximum viral population size after drug interruption. The larger the width between the set point and the maximum value of the viral population size, the higher the resonance [12]. Therefore, the resonance level can be simply quantified as the ratio between this maximum and the set point. This ratio summarises the measurements from the spectra given in [12], as it is shown in Figure 1.

### Parameters defining different viral loads

The resonance effect was proposed using the model given by Equations (1-3) [12] and using the parameters from a previous study [18]. Notably, the parameter values used vary significantly among different studies (see Table 1), resulting in a variation of the viral set point by several orders of magnitude, possibly reflecting the variation observed in empirical studies. Indeed, empirical studies have shown that the viral load at the set point varies enormously between patients and is correlated with the duration of the asymptomatic phase of HIV (and thus the development of AIDS) [19]. Therefore, the progression of the disease can be predicted, to some extent, by the set point. However, it is not clear whether the set point is related to the drug treatment failure.

**Table 1:** Different biological parameters used in the literature

**Parametrisation**	**Parameter values**	**Virus load at steady**	**References**
		**state (per day per mL)**	
I	*λ*=10, *d*=0.1, *a*=0.5		
	*u*=3, *β*=0.01, *k*=10	*v*^∗^=65.67	[15,20]
II	*λ*=20, *d*=0.02, *a*=0.4		
	*u*=4, *β*=5.10^−5^, *k*=100	*v*^∗^=850	[12,18]
III	*λ*=10^5^, *d*=0.1, *a*=0.5		
	*u*=5, *β*=2.10^−7^, *k*=100	*v*^∗^=3.5 10^6^	[15]
IV	*λ*=10^7^, *d*=0.1, *a*=0.5		
	*u*=5, *β*=5.10^−10^, *k*=500	*v*^∗^=1.8 10^9^	[13]

Table 1 summarises the set of parameters used by different authors and are classified here as parametrisation I, II, III, and IV according to their values. In particular, parametrisation II provided the basis for postulating the resonance effect as the cause of drug failure [12].

### Resonance level and oscillations

One of the main topics addressed in the present study is the relationship between resonance and oscillations. Specifically, we were interested in the possible link between the amplitude of the oscillations generated by the model after drug treatment is withdrawn (mainly the first peak of the virus rebound) and the degree of sustainability of the oscillations (i.e. the number of oscillations needed to approach the steady state). This last measure can be quantified as the ratio of the imaginary part and the real part of the complex eigenvalues, present in a system exhibiting damped oscillations [21]. Since the analytical expressions of the eigenvalues are too intricate to be studied, we simulated random variations of the parameters (taken from a uniform distribution) around their original value for each parametrisation I, II, III, and IV. If the original parameter value is *p*, then the new parameter values will be in a range *p*±*pα*, where *α*=0.7. The resulting parametrisations were selected to fulfill the following requirements: (1) positive equilibria of the populations involved (free virus, non-infected cells and infected cells), (2) negative real part of the eigenvalues for a stable equilibrium, (3) some of the eigenvalues of the Jacobian matrix of the system (1-3) should be complex, to ensure that the equilibrium is reached after damped oscillations [21].

A simple way to quantify the resonance level is to calculate the ratio between the maximum value of virus load after drug interruption and the viral set point. That is:

(4) $$\begin{array}{ll} R & =\frac{\text{max}(v(t))}{v^{*}}\; \end{array}$$

The value *R* therefore quantifies the resonance level in the virus population.

The degree of sustainability of the oscillations (*Ω*) is given by the relationship between the absolute value of the imaginary and the real part of the eigenvalues of the Jacobian matrix of the system (1-3):

(5) $$\begin{array}{ll} \Omega & =\frac{\left|\text{Im}(\text{Eigenvalues})\right|}{\left|\text{Re}(\text{Eigenvalues})\right|}\; \end{array}$$

**Figure 2:**
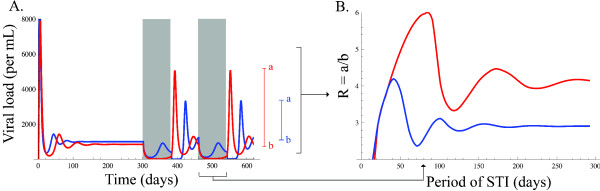
**Resonance level of random examples generated from parametrisation I (orange), II (red), III (green) and IV (blue).** Figure **A** shows the distribution of the samples generated for different viral set points, and the position of parametrisation II. Figure **B** shows the correlation between the degree of sustainability of the oscillations (*Ω*) and the resonance level (R).

Figure 2A and B, show the effect of the set-point virus load and of *Ω*, on the resonance level (*R*) for 10^4^ simulated patients. Consistent with Breban and Blower [12], we can see in Figure 2A that similar set point virus loads can exhibit different resonance levels. However, we also found that there is a tight correlation between the degree of sustainability of oscillations and the resonance level (Figure 2B). Therefore, according to the resonance hypothesis, drug failure should be observed in systems that exhibit sustained oscillations.

### Damped and sustained oscillations

The parametrisation II analysed in [12] has two important characteristics: a) soft damping, and therefore high resonance level (as showed in Figure 2) B, and b) small virus population size. When the population size is small, the effect of stochastic forces becomes more important. Evidence from other ecological and epidemiological systems have shown that models exhibiting damped oscillations in a deterministic approximation can present sustained oscillations driven by stochastic forces [22-25].

**Figure 3:**
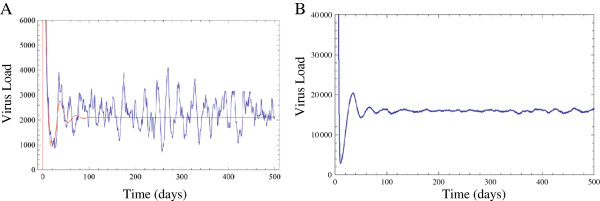
**Viral load over the time in the deterministic (red), and stochastic (blue) model. **. The parameters are: *λ*=20, *d*=0.02, *a*=0.4, *u*=4, *β*=5.10^−5^, *k*=200 (patient ‘C’ from [12]). For these parameters, stochastic dynamics induce sustained oscillations. **B**. For *λ*=2000, and changing *β* to maintain the same *R*_0_ (*β* = 0.05/(*λ*/*d*)), the oscillations are damped.

A full description of the process given by Equations (1-3) can be achieved considering each term as a probabilistic event (see Additional file 1). This formulation results in a master equation that can be expressed as a series expansion. The first order of the series corresponds to the deterministic system given by Equations (1-3). Higher orders become important when the population size is small (i.e. when the noise is high). In Figure 3, we show the effect of considering higher orders using a dynamic Monte Carlo simulation. We found that for the parameter values of parametrisation II, which result in a small viral set point, stochasticity induced sustained oscillations (Figure 3A). In Figure 3B we show that this effect depends on the population size: by changing *λ* and *β*, but keeping the basic reproductive ratio of the virus (*R*_0_) constant, we observed that the oscillations became damped. The difference between Figure 3A and 3B. can be described as follows: in deterministic systems, damped oscillations are characterised in the phase space by an unstable limit cycle (located in the vicinity of the first maximum and minimum values of the damping oscillations), and a stable equilibrium point (given by the steady state -in this case, known as the viral set point). The noise is higher in systems where the population size is small, and stochasticity effectively maintains the oscillatory regime around the unstable limit cycle. On the contrary, when the population size is large, stochasticity maintains the system far from the unstable limit cycle, and fluctuates instead in the proximity of the stable equilibrium point (the set point). This difference is important, since resonance is dependent on the distance between the maximum values attained by the viral population and the average values (set point). Both scenarios shown in Figure 3 are valid stochastic versions of the parametrisation II analysed in [12]. Which version (Figure 3A or Figure 3B) is considered more realistic, depends on the magnitude of stochastic effects in HIV, which is heavily debated [26].

### Parameters affecting viral rebound and limitations of the resonance hypothesis

**Figure 4:**
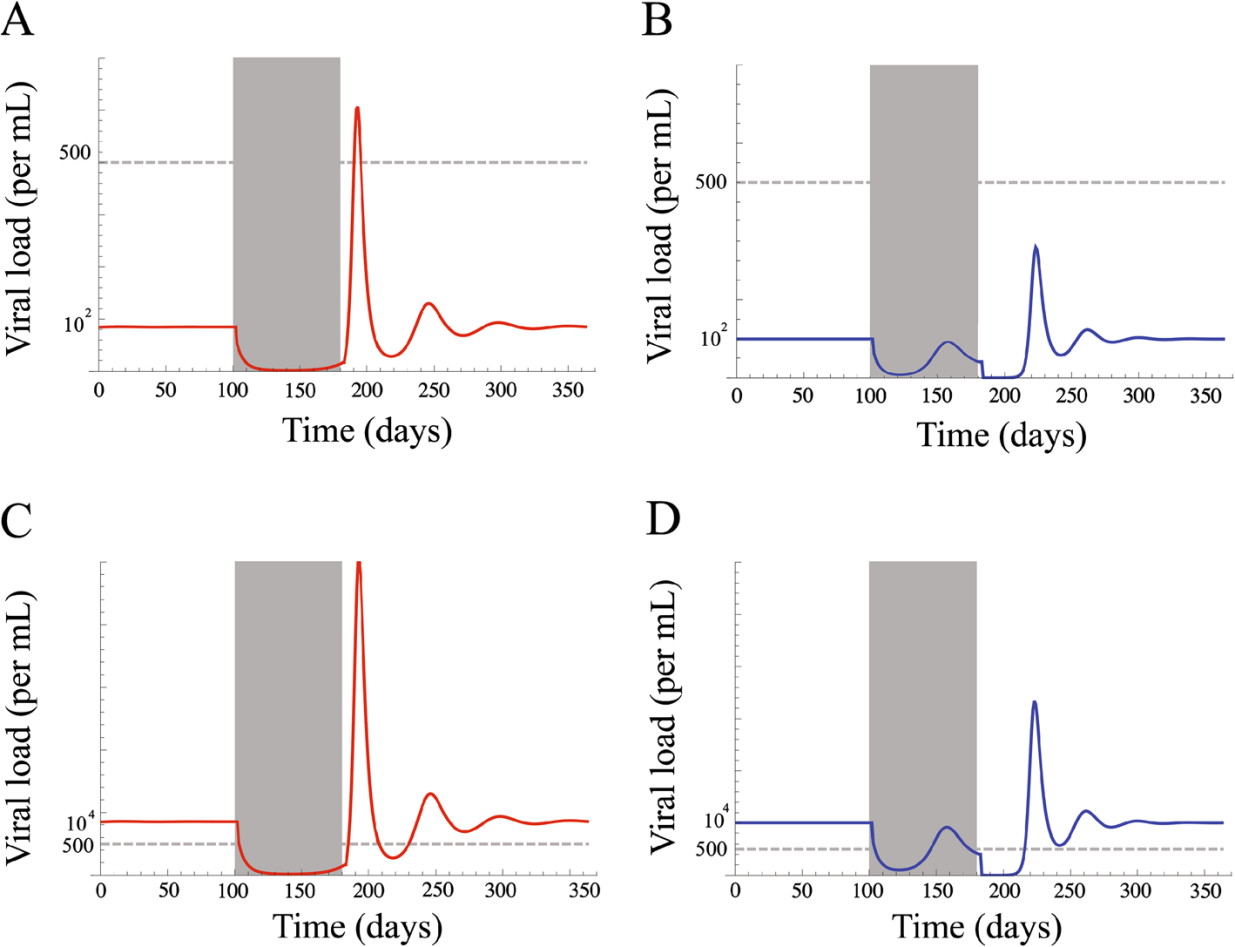
**Schematic illustration of the different possible relations between the viral set point and the failure threshold.** In Figure **A** and **B** the failure threshold is above the set point. In Figure **A** a strong resonant case generate a peak in viral load reaching the failure threshold. In Figure **B** the viral rebound do not reach the failure threshold. Figure **C** and **D** show a scenario where the failure threshold is below the set point. In this case the exponential viral rebound, crossing the failure threshold, determines the time of drug failure.

One could argue that the validity of the resonance hypothesis is restricted to an unrealistic parameter range. We considered two scenarios: failure threshold above the set point (Figure 4A and B) or failure threshold below the set point (Figure 4C and D). The first scenario is not plausible because, in the absence of drug therapy, the viral population at the set point is already below the failure threshold. Therefore, according to the treatment criteria chosen here, the patient should not have received therapy in the first place. Notably, this is the only scenario in which the resonance hypothesis holds. In a more realistic case, the failure threshold is below the viral set point (in fact, the empirical work upon which the resonance hypothesis is based, considers a failure threshold of 500 virus copies/ml [8]). As a consequence, the amplitude of the oscillations or the maximum value reached by the viral rebound does not affect drug failure. This indicates that drug failure is determined by the rate at which the viral population is growing during the exponential phase of viral rebound and, more specifically, how fast it reaches the “failure threshold”.

Although it is not possible to derive an analytical solution for the system given by differential Equations (1-3), an effective analytical approximation for the exponential phase of viral rebound can be obtained [15]. After drug interruption, the viral basic reproductive ratio is *R*_0_>>1. In these circumstances, the exponential phase of viral rebound after drug interruption can be approximated by:

(6) $$\begin{array}{l} v(t)=v_{0}e^{\frac{\mathrm{\beta \lambda k}}{\text{du}}t}\; \end{array}$$

The virus set point is given by:

(7) $$v^{*}=\frac{\mathrm{\lambda k}}{\text{au}}$$

From Equations (6) and (7) we derive two predictions. First, if two patients differ only in *λ*, *k* or *u*, we expect a correlation between *v*(*t*) and *v*^∗^. Second, if two patients differ only in *β*, *a* and *d*, then we expect no correlation between *v*(*t*) and *v*^∗^. These relationships explain why patients who have similar viral set points can have different responses to STI.

## Discussion

Resonance phenomena in biological systems are a dynamic area of research, with potential applications in different biological fields [27,28]. To our knowledge, the resonance hypothesis has been the only theoretical explanation proposed for drug failure under STI when such failure cannot be accounted for by viral resistance. The resonance hypothesis proposes that, after the interruption of drug therapy, the viral population reaches a maximum level, not correlated with the viral set point. Therefore, this hypothesis can account for cases in which two patients with similar set points at the outset, can result in STI failure in one patient and success in the other. The proponents of the hypothesis have acknowledged the speculative nature of the resonance hypothesis owing to the fact that viral oscillations have not been observed in HIV patients [12] –indeed, empirical data suggests that the pattern of virological decay and cumulative viral load after antiretroviral therapy in HIV-infected subjects is characterised by damped oscillation in the viral load [29] –. However, no detailed analysis has been made on the relationship between the proposed failure caused by the maximum of viral growth and the degree of sustainability of the viral oscillations. Also, it should be mentioned that even one single interruption in the drug administration produces the increase of the viral load, which indicates that the periodicity in drug administration does not seem to be necessary for the “resonance effect” to be observed.

Using a deterministic description of the model, we have shown that, for conditions in which the resonance effect is strong, oscillations are weakly damped (Figure 2B). We showed that a full description of the process, including stochastic effects, exhibited a sustained oscillatory regime for small viral population sizes (Figure 3A). Since weakly damped oscillations have not been observed in HIV-1 patients [14], the strong correlation between the degree of sustainability of oscillations and the resonance level suggests that the resonance hypothesis is not plausible. For some parameter regions, the model exhibits behaviour in line with empirical observations, namely a strong damping of oscillations. In these cases, the maximum viral rebound tends to be small. Finally we find that the viral peak reached after therapy interruption is attained after the viral population reaches the failure threshold. Therefore, in order to characterise the process that drives to drug failure, one should focus on the exponential rate of viral growth that occurs after drug interruption. The parameters of the model influencing this phase was presented and classified into those that affect the viral set point, those that affect the viral rebound rate, and those that alter both. We have shown that the basic model of virus dynamics can also account for the pattern of failure and success on patients with similar set points.

Further biological factors can influence the interaction between drug administration and HIV dynamics, as well as determine the viral set point. One important biological factor to consider is the cellular reservoir. HIV particles can remain in latent cells, conferring a reservoir for the virus that is not affected by drug treatment. The presence of a viral cellular reservoir will affect the plasma viral load to an extent that depends on the efficacy of the drug, and on the magnitude of latent cell activation [30]. A comparative approach using different types of models, which explicitly includes biological delays and latent cells, can be found in Holder and Beauchemin [31]. Another biological factor to consider is the immune response, which can play an important role determining the set point and the viral rebound rate. Including in the model spatial dynamics and delays in the interaction terms between cells and viral population can play a role in increasing or decreasing oscillations. However, we performed all analyses using the same model as the proponents of the resonance hypothesis. The rationale is that mathematical models provide a precise and explicit connection between assumption and conclusion. By including more complexity in the model, the assumptions and therefore the conclusions will be different. The use of the basic model has enabled us to provide a general picture of the pattern observed for HIV dynamics, and has allowed us to explore the consequences of the assumptions of the resonance hypothesis. In addition, it has provided an opportunity to analytically study the crucial parameters determining drug failure that occurs during the exponential phase of the viral rebound.

From a clinical perspective, our results explain how different patients can have similar initial viral set points but can differ in responses to therapy interruptions, without a resonance effect between the pattern of drug administration and the viral population dynamics. In fact, this effect is observable even in a single event of drug interruption. Therefore, given that the viral rebound caused by drug interruption does not require any periodicity in the drug administration, the mechanistic reason of the “treatment failure” described in the present study by viral rebound, can also be present as a consequence of low drug adherence. Therefore, although STI is not recommended in any patient infected with HIV, regardless of clinical status, the implications of the virological failure observed in empirical data from STI studies, along with the theoretical work that has emerged to explain the mechanistic basis of such failure, can also inform related topics, such as the consequences of low drug adherence. In this respect, further studies need to address questions regarding the effects of drugs with diverse kinetic parameters in combination with different levels of drug adherence on treatment failure by simple viral rebound or by the evolution of drug resistance [32].

## Conclusion

In the present study we combined deterministic and stochastic models of HIV infection to investigate the causes of drug failure observed in STI. We found evidence against the resonance hypothesis, which proposes that drug failure is caused by a resonance effect between the pattern of drug administration and the viral dynamics of HIV. We studied the parameters affecting the viral set point and viral rebound. Our analysis enabled us to establish the crucial parameters which determine the drug failure that occurs during the exponential phase of the viral rebound.

## Competing interests

The authors declare that they have no competing interests.

## Authors’ contributions

Conceived, designed and performed the experiments: LO, RK, ML, SB. Developed the mathematical models: LO, RK. Wrote computer simulation and analysis programs: LO, RK. Analysed the data: LO. Wrote the paper: LO, RK, SE. All authors read and approved the final manuscript.

## Supplementary Material

Additional file 1 **Supporting information **[33]**.** Click here for file
