# Supplementary material for: On the role of resonance in drug failure under HIV treatment interruption
Source: Theor Biol Med Model. 2013 Jul 11;10:44. doi: 10.1186/1742-4682-10-44 (PMC3718686; doi:10.1186/1742-4682-10-44)
Supplement: Additional file 1 — Supporting information [33]. [file 1742-4682-10-44-S1.pdf]

Additional file

Supporting Information

It is possible to re-write the equations (1-3), considering each interaction term as a probabilistic event:

$$A \overset{\lambda}{\rightarrow} x \quad (1)$$
$$y \overset{k}{\rightarrow} v \quad (2)$$
$$x + v \overset{\beta}{\rightarrow} y + v \quad (3)$$
$$x \overset{d}{\rightarrow} 0 \quad (4)$$
$$y \overset{a}{\rightarrow} 0 \quad (5)$$
$$v \overset{u}{\rightarrow} 0 \quad (6)$$

A is necessary for a virus birth event to take place and through (8), A is a reservoir, used to model a constant influx into the system [33]. The direct interaction of a susceptible cell with a virus particle in (3) result in the generation of an infected cell at a rate β. The reaction rates, in these cases, must satisfy some requisite, as will be discussed later. The system state in a given time is described by the multivariant probability density function $P(x, y, v, t)$, of the stochastic variables x, y and v. The transitions between different states are given by the processes (1-6). The transition probabilities must be calculated for all the implied processes in order to write the master equation that governs the evolution of $P(x, y, v, t)$. These transition probabilities will be written finishing in the status (x, y, v), which may also be written without problem, but they will not be necessary in the following proposal:

$$T(x+1, y, v \mid x, y, v) = \lambda \quad (7)$$
$$T(x, y, v+1 \mid x, y, v) = y\ k \quad (8)$$
$$T(x-1, y+1, v \mid x, y, v) = 2\,x\ v\ \beta \quad (9)$$
$$T(x-1, y, v \mid x, y, v) = x\ d \quad (10)$$
$$T(x, y-1, v \mid x, y, v) = y\ a \quad (11)$$
$$T(x, y, v-1 \mid x, y, v) = v\ u \quad (12)$$

Notice that there is a factor 2 in the probabilities of the processes that imply two elements of different populations, such as $x + v$, which is not present in events that imply reactions of only one cell (deaths) or in events that imply entities of the same population. In this case we consider the entities of a population as undistinguishable, effectively as molecules of a chemical system. The master equation for $P(x, y, v, t)$ is read in general as:

$$\frac{dP(x,y,v)}{dt} = \sum_{i,j,k} T(x, y, v \mid x_i, y_j, v_k)\, P(x_i, y_j, v_k) - \sum_{i,j,k} T(x_i, y_j, v_k \mid x, y, v)\, P(x, y, v) \quad (13)$$

where the sum is taken over all the states allowed by the transitions to or from the state (x, y, v) listed below. The form of the equation becomes more manageable in terms of step operators defined as acting in the state variables:

$$\epsilon_x f(x) = f(x+1) \quad (14)$$
$$\epsilon_y f(y) = f(y+1) \quad (15)$$
$$\epsilon_v f(v) = f(v+1) \quad (16)$$

and their inverse:

$$\begin{aligned}\epsilon_x^{-1} f(x) &= f(x-1) &(17)\\ \epsilon_y^{-1} f(y) &= f(y-1) &(18)\\ \epsilon_v^{-1} f(v) &= f(v-1) &(19)\end{aligned}$$

With these operators the following is obtained:

$$\begin{aligned}\frac{dP(x,y,v)}{dt} &= [(\epsilon_x^{-1}-1)T(x+1,y,v \mid x,y,v)\\ &+(\epsilon_v^{-1}-1)T(x,y,v+1 \mid x,y,v)\\ &+(\epsilon_x\epsilon_y^{-1}-1)T(x-1,y+1,v \mid x,y,v)\\ &+(\epsilon_x-1)T(x-1,y,v \mid x,y,v)\\ &+(\epsilon_y-1)T(x,y-1,v \mid x,y,v)\\ &+(\epsilon_v-1)T(x,y,v-1 \mid x,y,v)]P(x,y,v) \qquad (20)\end{aligned}$$

Expansion of the Master equation

The systematic expansion of the master equation developed by van Kampen [33] lies in the following ansatz, that decomposes each stochastic variable into a macroscopic and analytical variable (in this case: ϕ for x, ψ for y and Ω for v), plus a small amplitude noise given by a random variable (in this case: ξ for x, η for y and θ for v) scaled by $N^{1/2}$:

$$\begin{aligned}x &= N\phi+\sqrt{N}\xi &(21)\\ y &= N\psi+\sqrt{N}\eta &(22)\\ v &= N\Omega+\sqrt{N}\theta &(23)\end{aligned}$$

Here $N = x + y + v$. The step operators have simple expansions in powers of $\sqrt{N}$, which are particularly useful. For example:

$$\begin{aligned}\epsilon_x &= 1+\frac{1}{\sqrt{N}}\frac{\partial}{\partial\xi}+\dots &(24)\\ \epsilon_x^{-1} &= 1-\frac{1}{\sqrt{N}}\frac{\partial}{\partial\xi}+\dots &(25)\end{aligned}$$

And similarly for ϵ_i.

Using (21-25) in the master equation an expansion in powers of $\sqrt{N}$ is obtained which may be analysed order to order. This systematic development of the master equation makes possible an analytical approximation to the stochastic problem. In particular, we are interested in analysing the validity of the mean-field equations, both for big and small systems. On the other hand, there are inherently stochastic phenomena whose analytical treatment requires one or another form of resolution of the master equation (or its computational implementation). In the next sections the development of van Kampen for (20) system will be implemented and some of these phenomena will be briefly analysed.

Macroscopic Equations

Replacing by the expression in the way of expansion in powers of $\sqrt{N}$ of the step operators and the transition probabilities (7 - 10), the master equation may be written as:

$$\begin{aligned}
\frac{dP(x,y,v)}{dt} &= \left(-\frac{1}{\sqrt{N}}\frac{\partial}{\partial\xi}+\frac{1}{2N}\frac{\partial^2}{\partial\xi^2}\right)\lambda\,\Pi \\
&+ \left(-\frac{1}{\sqrt{N}}\frac{\partial}{\partial\theta}+\frac{1}{2N}\frac{\partial^2}{\partial\theta^2}\right) y\,k\,\Pi \\
&+ \left[\left(1+\frac{1}{\sqrt{N}}\frac{\partial}{\partial\xi}+\frac{1}{2N}\frac{\partial^2}{\partial\xi^2}\right)\left(1-\frac{1}{\sqrt{N}}\frac{\partial}{\partial\eta}+\frac{1}{2N}\frac{\partial^2}{\partial\eta^2}\right)-1\right] 2\,\tilde{\beta}\,x\,v\,\Pi \\
&+ \left(\frac{1}{\sqrt{N}}\frac{\partial}{\partial\xi}+\frac{1}{2N}\frac{\partial^2}{\partial\xi^2}\right) d\,x\,\Pi + \left(\frac{1}{\sqrt{N}}\frac{\partial}{\partial\eta}+\frac{1}{2N}\frac{\partial^2}{\partial\eta^2}\right) a\,y\,\Pi \\
&+ \left(\frac{1}{\sqrt{N}}\frac{\partial}{\partial\theta}+\frac{1}{2N}\frac{\partial^2}{\partial\theta^2}\right) u\;v\;\;\Pi + \ldots \\
&= \frac{\partial\Pi}{\partial t} - \sqrt{N}\left(\dot{\phi}\frac{\partial\Pi}{\partial\xi}+\dot{\psi}\frac{\partial\Pi}{\partial\eta}+\dot{\omega}\frac{\partial\Pi}{\partial\theta}\right) \qquad (26)
\end{aligned}$$

The dots in equation (26) indicate the terms of the expansion of the step operators that were not taken into account.

It is possible to group the expression (26) in terms $\sqrt{N}$, $\sqrt{N^0}$ and terms of order $\sqrt{N^{-1}}$, $\sqrt{N^{-2}}$..., of the form:

$$\begin{aligned}
\frac{dP(x,y,v)}{dt} &= \sqrt{N}\left[\frac{\partial\Pi}{\partial\xi}(-\lambda+2\tilde{\beta}\phi\omega+d\phi)+\frac{\partial\Pi}{\partial\eta}(-2\tilde{\beta}\phi\omega+a\psi)\right. \\
&+ \left.\frac{\partial\Pi}{\partial\theta}(u\omega-k\psi)\right] \qquad (27) \\
&+ \sqrt{N^0}\left[\frac{\partial\Pi}{\partial\xi}\left(2\tilde{\beta}(\phi\theta+\xi\omega)+d\xi\right)+\frac{\partial^2\Pi}{\partial\xi^2}\left(\frac{1}{2}\lambda+\tilde{\beta}\phi\omega+\frac{1}{2}d\phi\right)\right. \\
&+ \frac{\partial\Pi}{\partial\eta}\left(-2\tilde{\beta}(\phi\theta+\xi\omega)+\eta\right) \\
&+ \frac{\partial^2\Pi}{\partial\eta^2}\left(\tilde{\beta}\phi\omega+\frac{1}{2}a\right)+\frac{\partial\Pi}{\partial\theta}(-k\eta+u)+\frac{\partial^2\Pi}{\partial\theta^2}\left(\frac{1}{2}u\,\omega\right) \\
&+ \left.\frac{\partial^2\Pi}{\partial\xi\partial\eta}\left(\tilde{\beta}\phi\omega\right)\right] \qquad (28) \\
&+ O\left[\frac{1}{\sqrt{N}}\right] \qquad (29) \\
&+ O\left[\frac{1}{N}\right]+\ldots \qquad (30)
\end{aligned}$$

In turn, by reordering the terms of order $\sqrt{N}$, given by (28) the following is obtained:

$$\begin{aligned}
&\sqrt{N}\left\{[\dot{x}+\lambda-dx-2\tilde{\beta}xv]\frac{\partial\Pi}{\partial\xi}\right. \\
&\left.+[\dot{y}+2\tilde{\beta}xv-ay\,]\frac{\partial\Pi}{\partial\eta}+[\dot{v}+ky-uv]\frac{\partial\Pi}{\partial\theta}\right\} \qquad (31)
\end{aligned}$$

and in order to be able to cancel at each time the terms that accompany $\frac{\partial\Pi}{\partial\xi}$, $\frac{\partial\Pi}{\partial\eta}$ and $\frac{\partial\Pi}{\partial\theta}$ (the coefficients

of it should occur that:

$$\dot{x} = \lambda - dx - 2\tilde{\beta}xv \tag{32}$$

$$\dot{y} = 2\tilde{\beta}xv - ay \tag{33}$$

$$\dot{v} = ky - uv \tag{34}$$

By taking $\beta = 2\tilde{\beta}$ one obtains the macroscopic equations (1- 3).
